# Integrating the Prevention and Control of Rheumatic Heart Disease into Country Health Systems: A Systematic Review and Meta-Analysis

**DOI:** 10.5334/gh.874

**Published:** 2020-09-14

**Authors:** Jessica Abrams, David A. Watkins, Leila H. Abdullahi, Liesl J. Zühlke, Mark E. Engel

**Affiliations:** 1 Department of Paediatrics and Child Health, University of Cape Town, ZA; 2 Department of Medicine, University of Washington, Seattle, WA, US; 3 Department of Paediatrics, Red Cross War Memorial Children’s Hospital, University of Cape Town, ZA; 4 Department of Medicine, Groote Schuur Hospital and Faculty of Health Sciences, University of Cape Town, ZA; 5 Department of Medicine, University of Cape Town, ZA

**Keywords:** integration, rheumatic fever, rheumatic heart disease

## Abstract

**Background:**

National and international political commitments have been made recently on rheumatic heart disease (RHD), a preventable heart condition that is endemic in low-resource countries. To inform best practice and identify evidence gaps, we assessed the effectiveness of RHD prevention and control programmes and the extent and nature of their integration into local health systems.

**Methods:**

We conducted a systematic review and meta-analysis using a previously published protocol that included electronic and manual searches for studies published between January 1990 and July 2019 reporting on prevention and control programmes for populations at risk for streptococcal pharyngitis, rheumatic fever, and/or RHD. We analysed programme integration according to a previously published framework and programme effectiveness using a results-chain framework. We meta-analysed secondary prophylaxis adherence using random-effects models. Study quality was assessed using peer-reviewed checklists (CASP and PRISM). PROSPERO registration: CRD42017076307.

**Findings:**

Five observational studies met with the inclusion criteria. Studies were similar in extent and nature of integration into health systems; no programme was completely integrated or non-integrated. A single study reported on programme impact. Secondary prophylaxis adherence improved among partially integrated RHD programmes (RR, 1.18 [95% CI, 1.03 to 1.36], 3 studies, n = 618). Risk of bias was low in two studies, and indeterminable in the remaining three studies.

**Interpretation:**

There is evidence that partially integrated RHD programmes are beneficial for a range of intermediate health outcomes. This review provides a starting point for the design and implementation of future RHD programmes by outlining current best practice for integration and identifying key gaps in knowledge.

**Funding:**

National Research Foundation of South Africa.

## Introduction

Rheumatic heart disease (RHD) is a potentially fatal yet preventable condition which begins with a sore throat and results in damage to the valves of the heart. RHD is responsible for about 300,000 deaths annually, most of which are children and young adults from resource-constrained settings [1]. Crowded and unsanitary living conditions enable the spread of group A streptococcus (Strep A), the infectious agent inducing an autoimmune response, resulting in the progression from pharyngitis to acute rheumatic fever (ARF) [2]. Under-recognition of ARF coupled with inadequate access to medical care often results in RHD and sometimes premature death among these patients [2].

Strategies to combat disease progression include penicillin primary prophylaxis following Strep A diagnosis, or secondary prophylaxis for patients diagnosed with ARF or RHD [2,3]. In patients who present for medical attention late in the disease, heart valve surgery is usually required to repair the damage caused by severe or recurrent episodes of ARF, often followed by a lifelong dependence on anticoagulants and penicillin [4]. In countries with endemic patterns of RHD, weak infrastructure and limited resources are key barriers to RHD prevention and control efforts [5].

RHD has been placed on the international agenda, with the World Heart Federation setting out to achieve a 25% reduction in premature deaths from ARF and RHD among individuals younger than 25 years of age, by the year 2025 [6,7]. More recently, the World Health Assembly approved the Resolution on Rheumatic Fever and Rheumatic Heart Disease thereby committing countries to showing progress in the eradication of RHD [8]. The Addis Ababa Communique and Cairo Accord provide key actionable strategies to eradicate RHD which includes implementing a multi-sectoral national RHD programme [9,10].

In order to achieve the desired progress, evidence-based prevention and treatment services require scaling up in the countries and regions which are still heavily burdened with RHD. Technical experts recommend comprehensive prevention and control programmes that are integrated into country health systems [5], as integrated health interventions have been found to have health system strengthening features [11,12]. By contrast, a number of historical examples of RHD programmes appear to have been run as standalone initiatives, and their claims of effectiveness were based on study designs that lacked appropriate controls [13].

The 2018 World Health Assembly resolution and resulting political commitment to tackle RHD have added urgency to the need for technical guidance on the design of RHD control programmes that can be integrated within primary healthcare and universal health coverage systems in low-resource settings [14]. In the present study, we conduct a systematic review and meta-analysis to examine published reports of RHD prevention and control programmes in order to determine the nature and extent of integration according to six key health system functions, and how this integration (or lack thereof) might affect programme success.

## Methods

### Overview

To assess the integration of published examples of RHD control programmes into country health systems, we drew on a conceptual model of integration that was developed by Atun and colleagues [15]. They noted that ‘integration’ is not an all-or-nothing characteristic of a programme; depending on the various functions of a programme (e.g., financing, monitoring and evaluation), it can be characterised as more- or less-integrated into the general health system of a country. In a separate paper, Atun and colleagues also demonstrated the variation in the degree of integration of various programmes for child health and communicable diseases, and they stressed the lack of evidence that more integrated programmes were always more effective [16].

The Preferred Reporting Items for Systematic Reviews and Meta-analyses (PRISMA) guidelines were followed in this systematic review (Appendix 1) [17]. Further details on the study protocol have been published elsewhere (https://bmjopen.bmj.com/content/bmjopen/9/6/e028908.full.pdf) [18]. This study was registered with PROSPERO, number CRD42017076307.

### Search strategy and inclusion criteria

We searched PubMed, Cochrane Central Register of Controlled Trials, Scopus, ISI Web of Science, Africa Wide and CINAHL for published studies using a comprehensive search strategy (Appendix 2). Google Scholar and Global Index Medicus (which includes Latin America and the Caribbean database LILACS, as well as World Health Organization Library Information System (WHOLIS)) were searched for grey literature using key search terms. The reference lists of relevant studies were hand searched to further identify possible articles. Studies were eligible for screening if they were published in English between 1 January 1990 and 31 July 2019.

After removing duplicate publications, two authors (JA and DW) independently screened titles and abstracts. Thereafter the same authors independently screened the full-text articles to determine inclusion. Discrepancies were resolved by discussion and consultation with a third author (ME) when necessary. As described in our previously published protocol, studies were included if they reported on a health programme (defined as an intentional effort to expand health services) directed at populations at risk for Strep A infection, ARF, and/or RHD. In addition, studies had to provide sufficient information on programme characteristics such as the duration and location of the programme, the type of services delivered, and the programme inputs, as well as details on at least four of the six key functions of the health system, namely: (i) governance, (ii) financing, (iii) planning, (iv) service delivery, (v) monitoring and evaluation, and (vi) demand generation. Randomised controlled trials (RCTs), controlled clinical trials (CCTs), quasi-experimental, controlled before-and-after studies (CBAs), interrupted time series (ITS), or cross-sectional designs (i.e., programme case reports) were included. ‘Opinion pieces’, narrative reviews, and letters to the editor were excluded.

### Data analysis

Using a piloted data extraction form, we extracted a variety of quantitative and qualitative data related to the programme characteristics, extent of integration, and programme results. Programme characteristics included basic data such as target population, scale, and duration, as well as detailed data on inputs organised into the six ‘building blocks’ used in the WHO health systems framework. The extent of integration was characterised for each of the six key functions of the health system and assigned a score from 1 to 3 depending on whether the programme was not integrated, partially integrated, or fully integrated (or not reported). Integration scores for each of the six key functions were summed to a composite score with a maximum value of 18 (see Appendix 3 for further details on scoring criteria). A results chain comprising programme inputs, activities, outputs, outcomes, and impacts, was populated for each study using the extracted data. A random-effects meta-analysis of study outcomes was performed using Review Manager 5.3 [19]. Data were either pooled or presented without totals depending on the model of care delivery and type of outcome.

The first six domains of the Critical Appraisal Skills Programme (CASP) checklist were used to assess the risk of bias of the included studies [20]. Domains were scored as ‘Y’ (bias absent), ‘N’ (bias present), not applicable, or unclear.

### Role of the funding source

The sponsor had no role in study design, data collection, data analysis, data interpretation, writing of the report, or the decision to submit the paper for publication. All authors has full access to all the data in the study and had responsibility for the decision to submit for publication.

## Results

**Figure 1:**
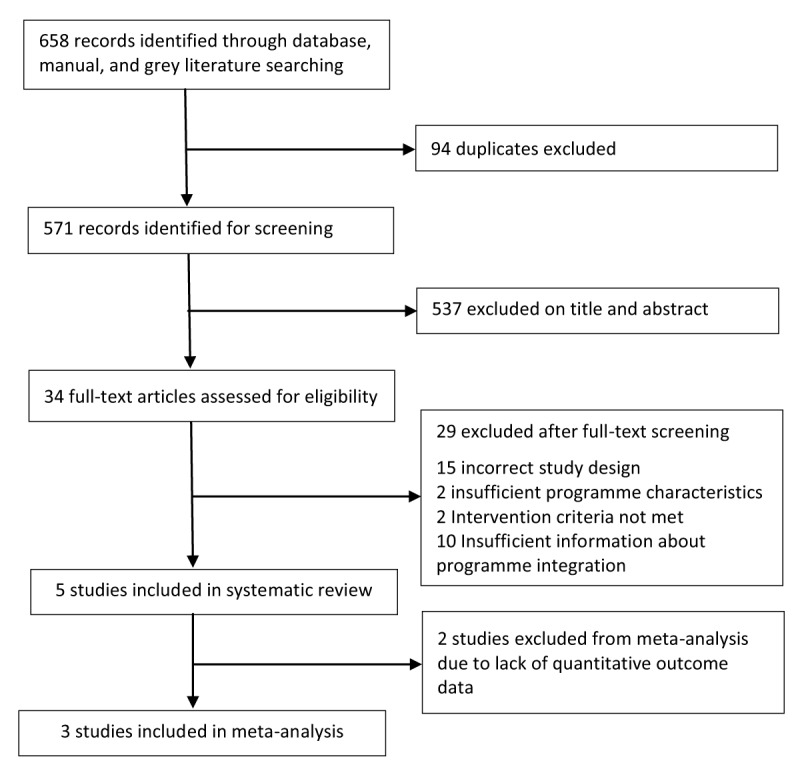
Study selection.

The search identified 658 publications, of which 94 were duplicates, leaving a total of 564. An additional seven articles were found following grey literature and reference list searches. During title and abstract screening, 537 studies were excluded. The remaining 34 publications underwent detailed assessment; a further 29 articles were excluded, mostly because of unacceptable study design or insufficient information on programme integration (Figure 1). Five articles were included in this systematic review [21,22,23,24,25], characteristics for which are shown in Table 1.

**Table 1:** Characteristics of included studies (ordered chronologically).

Study ID	Country and region	Programme duration	Description of the intervention	Study outcome(s) measured	Level(s) of prevention or care			Programme scale: numbers of healthcare workers and patients involved

					1°	2°	3°	

Iyengar 1991 [21]	India: Haryana State, Ambala district.	2 years	An ARF/RHD health education and training programme for health workers, teachers, and school pupils, as well as the registration of new cases and prescription of penicillin.	I. The number and source of: suspected case referrals, registered cases, and confirmed cases of RF and RHD (case detection rate) II. Adherence to secondary prophylaxis.		✓		202 healthcare workers and 773 teachers were trained to recognise the signs and symptoms of ARF and RHD. Of the 254 suspected case referrals, 77 were registered in health centres, of which 61 were confirmed and began secondary prophylaxis.
WHO 1992 [23]	16 countries participated: (Africa) Mali, Zambia, Zimbabwe; (Americas) Bolivia, El Salvador, Jamaica; (Eastern Mediterranean) Egypt, Iraq, Pakistan and Sudan; (South-East Asia) India, Sri Lanka and Thailand; (Western pacific) China, the Philippines, and Tonga.	4 years	Personnel training, health education and a central ARF/RHD register.	I. Secondary prophylaxis coverage. II. ARF reoccurrence.		✓		Across all of the countries, 24 398 personnel trained; 33 651 patients were registered.
Nordet 2008 [22]	Cuba: Pinar del Rio.	10 years	A community based prevention and treatment of ARF/RHD through healthcare education and training of health personnel as well as the establishment of dedicated register centres.	I. The incidence of ARF (new and recurrent cases). II. The prevalence and severity of RHD. III. Secondary prophylaxis compliance. IV. The proportion of patients requiring hospitalization.	✓	✓		All 5–25 year old permanent residents of the province during the study period were included (n = 273 933).
Ralph 2013 [24]	Australia: Northern Territory.	3 years	A continuous quality improvement (CQI) strategy to improve the documentation and care of ARF/RHD patients.	I. Proportion of patients receiving scheduled BPG. II. Proportion of patients reviewed by their doctor in the past two years. III. The quality of data recorded on ARF/RHD patients: ARF episodes and RHD risk category information.		✓		6 health centres participated; 154 ARF/RHD patients.
Kwan 2013 [25]	Rwanda: Kirehe and Southern Kayonza districts.	4.4 years	Outpatient heart failure services implemented at pre-existing integrated NCD clinics at two rural hospitals. Portable ECG and algorithms were used for the diagnosis and management of patients with suspected heart failure.	I. Distribution of conditions (including RHD) among heart failure patients. II. Programme retention. III. Mortality among patients with confirmed diagnoses.			✓	Each clinic team included 2 nurses and 2 administrative personnel, supervised by generalist physicians. Out of 237 patients suspected of heart failure, 192 had a confirmed cardiologist diagnosis and were enrolled in the heart failure programme.

ARF, acute rheumatic fever; RHD, rheumatic heart disease.

### Characteristics of included studies

All five included studies were observational; three used a cross-sectional design while the remaining two used a quasi-experimental (before and after) study design. Four of the five studies focused on secondary prevention of RHD [21,22,23,24], though one also had a primary prevention component [22]. A single study targeted tertiary care [25].

The outcomes measured varied across the studies with none of the studies specifically assessing approaches to integrating RHD care as a primary study objective. A variety of geographical locations were covered by the included studies, targeting at-risk communities in Africa, the Americas, South-East Asia, the Eastern Mediterranean, and the Western Pacific region. The shortest study duration was two years while the longest study continued for 10 years.

### Programme inputs and activities

Each of the studies described their programme inputs (financial, human, and material resources) and activities (mobilisation of inputs) to varying degrees of detail. Financing and personnel inputs were well described across the studies, but activities such as systems of clinical care delivery were underreported, making it difficult to replicate the programme. A full description of programme inputs and activities of the included studies are provided in Appendix 4.

### Extent of Integration

**Figure 2:**
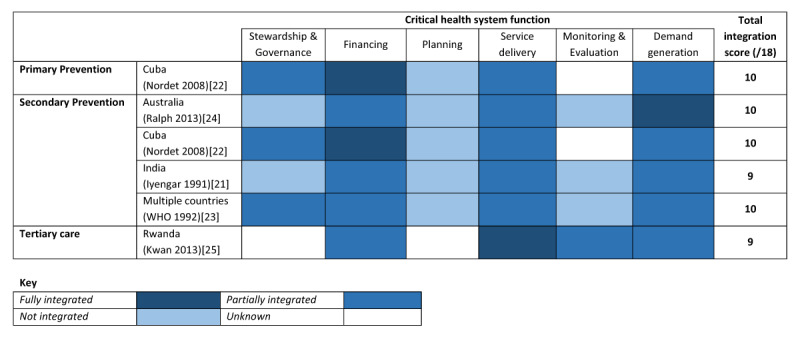
The extent and nature of integration by level of prevention for rheumatic heart disease programmes in various countries.

The extent (fully, partially, not, unknown) and nature (type of health system function) of integration for the RHD prevention and control programmes included in this study are described in Figure 2. The composite programme integration score was similar across all of the studies (either 9 or 10 out of 18), meaning that none of the programmes were completely integrated into the health system across all key functions.

Financing was most often partially, if not fully, integrated across all of the programmes. Funds which did not come from the general health budget were supplemented by external organisations (such as AGFUND) but were channelled through the health system. Service delivery and demand generation were the second most integrated health system functions. The service delivery model employed by primary and secondary programmes were similar, where prophylaxis was administered in primary health care settings together with other health services, while case-finding efforts were more targeted. Similarly, demand generation was often executed separate from other health education activities (i.e., dedicated campaigns on RHD) but undertaken as part of the local government activities.

Stewardship and governance was either partially or not integrated. Ministries of Health contributed to the governance of several programmes, but often accountability lay solely with a dedicated entity (either within the ministry or within an academic medical institution). Programme planning and monitoring and evaluation were almost never integrated into the health system among these programmes. Public health sector employees were usually involved in planning, but decision-making appeared to focus on RHD alone and did not consider other aspects of the health system or other diseases. There were insufficient details about stewardship and governance and programme planning for the Rwandan tertiary care intervention. Monitoring and evaluation was not well described in the primary and secondary programme implemented in Cuba.

### Programme Performance: Outputs, Outcomes and Impact

Each study reported on slightly different programme outcomes; only the Cuban study presented evidence of the impact of their programme on disease endpoints (incidence, prevalence, and mortality) (Table 2). The primary and secondary programmes in Cuba resulted in fewer recurrent and first ARF attacks, and there was an overall decline in the prevalence of ARF and RHD. The severity of RHD was also controlled with fewer patients requiring hospitalization [22]. In Australia’s Northern Territory, there was a statistically significant improvement in the details documented on ARF and RHD patients, but the overall proportion of patients receiving ≥80% of scheduled BPG did not improve [24]. The programme in India successfully improved the ARF and RHD case detection rate in a high-risk community (from 7.8 cases per 100 000/year to 27.5 cases per 100 000/year), and registered patients maintained an 85-95% secondary prophylaxis compliance [21]. The average rate of prophylaxis coverage was 70% in the RHD programme implemented by WHO in multiple countries [23]. The tertiary care clinic in Rwanda saw 61 patients with RHD, of which 3 died, over the course of 4.4 years [25].

**Table 2:** Programme performance.

Country (Study ID)	Outputs	Outcomes	Impact

**Primary prevention**			

**Cuba (Nordet 2008) [22]**	▪ Increased medical awareness among young patients.	▪ Timely diagnosis and treatment of strep-throats.	▪ The incidence of first ARF attacks declined from 12.2 per 100 000 in 1986 to 2.1 per 100 000 in 1996.
**Secondary prevention**			

**Australia (Ralph 2013) [24]**	▪ The number of clinical records audited each year were 154 in 2008, 145 in 2009, and 156 in 2010.	▪ The proportion of patients receiving ≥40% of scheduled BPG increased from 81/116 (70%) at baseline to 84/103 (82%) in year three, p = 0.04. ▪ The proportion of people receiving ≥80% of scheduled BPG did not improve, remaining around 25% across all six health centres over the study duration. ▪ More patients were reviewed by their doctor within the past two years: from, 112/154 (73%) to 134/156 (86%), p = 0.003. ▪ Improved details on patients with ARF/RHD: ARF episode documentation increased from 31/55 (56%) to 50/62 (81%) (p = 0.004), and RHD risk category documentation from 87/154 (56%) to 103/145 (76%) (p < 0.001). ▪ Patients within the recommended INR range increased from 64% to 75%.	▪ *Not reported*
**Cuba (Nordet 2008) [22]**	▪ 327 patients registered over the study period.	▪ Increased regular secondary prophylaxis compliance of registered patents (from 50% in 1986 to 93.8% in 1996). ▪ 86.1% decline in the cost of managing the disease.	▪ Decline in the prevalence of ARF and RHD (8.0 to 2.0 cases per 1 000 school children). ▪ Decline in the incidence of recurrent attacks of ARF (6.4 to 0.4 per 100 000). ▪ Decreased severity of RHD (5 cases of severe RHD in 1986 to only 1 in 1996). ▪ Decrease in the number and of patients requiring hospitalization after the acute attack (from 41.1% of the 134 registered cases during 1986-90 to 8.3% of the 193 registered cases during 1991–96).
**India (Iyengar 1991)[21]**	▪ A total of 254 suspected cases of ARF or RHD referred by teachers, health workers, and medical officers.	▪ 3.5 time increases in the case detection rate in the intervention block (7.8/100 000/year to 27.5/100 000/year). ▪ 95% compliance to secondary prophylaxis in the first 6 months, this declined to 85% after 2 years.	▪ *Not reported*.
	▪ The diagnosis and registration of 77 new cases of ARF/RHD (of which 61 were subsequently confirmed to have the disease).		
**Multiple countries (WHO 1992) [23]**	▪ 33 651 total patients identified and registered. ▪ 95.7% of patients received BPG injections, 2.1% oral penicillin, 0.1% sulfadiazine, and 2.1% erythromycin. ▪ 36 patients had an adverse reaction to BPG (0.3% patient-years), of whom 4 died.	▪ The rate of average prophylaxis coverage was 70%. ▪ The rate of coverage per 100 patients registered per month averaged 63.2% (range, 23.8–96.9%). ▪ Reoccurrence of ARF occurred in 53 patients (0.4% patient-years), of whom only 2 were receiving regular BPG.	▪ *Although it is stated that the reoccurrence rate of ARF decreased, no evidence was presented*.
**Tertiary care**			

**Rwanda (Kwan 2013) [25]**	▪ 192 patients were confirmed to have heart failure and were enrolled at the clinic. Of this cohort, 61 patients (32%) had RHD (26 patients were below the age of 18 years and 35 patients were adults). ▪ Over the course of 4.4 years, the mean time spent in care was 19 months. The median time in care for alive patients with complete records (n = 169) was 13 months for children and 20 months for adults.	▪ The observed retention in the programme was 62%. Fifty-five patients (29%) were lost to follow-up. ▪ 18 patients (9%) died, of which 3 had RHD. Mortality might be underestimated due to those lost to follow-up.	▪ *Not reported*.

### Acute Rheumatic Fever and Rheumatic Heart Disease-Related Outcomes

Overall, programmes that are at least partially integrated in several dimensions appear to have a positive effect on clinical outcomes (Figure 3A). Specifically, improvements in the following outcomes were documented: incidence of first ARF attacks (RR, 0.08 [95% CI, 0.02 to 0.33]), recurrent ARF attacks (RR, 0.22 [95% CI, 0.07 to 0.76]), hospitalization rates following an AFR attack (RR, 0.22 [95% CI, 0.00 to 0.15]), rates of severe RHD (RR, 0.05 [95% CI, 0.01 to 0.45]), prevalence of ARF and RHD (RR, 0.24 [95% CI, 0.16 to 0.36]), and patients out of INR range (RR, 0.70 [95% CI, 0.23 to 2.11]). All of the outcomes were statistically significant, except for patients out of INR range which contains the null value of one in the 95% confidence interval.

**Figure 3A:**
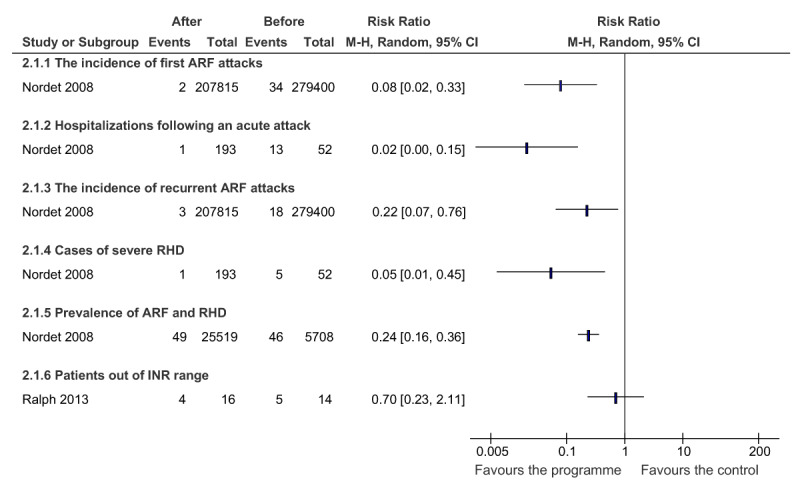
The effect of partially integrated ARF/RHD programmes on ARF/RHD-related outcomes.

### Meta-analysis: Acute Rheumatic Fever Secondary Prophylaxis Compliance

Data on secondary prophylaxis were amenable to meta-analysis. Three studies defined secondary prophylaxis compliance as the probability of a patient receiving ≥80% of administered prophylaxis on a regular basis. There was a significant improvement in secondary prophylaxis compliance (RR, 1.18 [95% CI, 1.03 to 1.36], 3 studies, n = 618) amongst patients subjected to a partially integrated programme (Figure 3B).

**Figure 3B:**
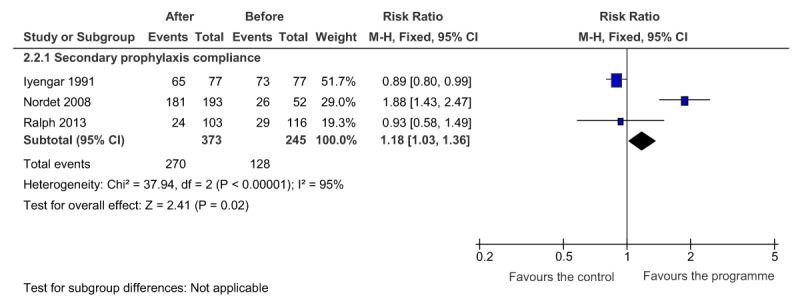
The effect of an integrated programme on ARF secondary prophylaxis compliance.

Overall, two studies were found to have a low risk of bias while the remaining three studies were unclear (Appendix 5). In two of the studies, it was unclear whether the outcome measure was accurately measured to minimise bias. One study inadequately described their method of cohort recruitment.

## Discussion

This systematic review provides the first structured assessment of the extent of integration of RHD programmes into country health systems. We also collected information on programme inputs, activities, outputs, outcomes, and impact, but due to the limitations in the designs of the included studies we were not able to assess the association between programme design, programme integration, and population health outcomes. While most of the RHD programmes specified different outcomes, each demonstrated improved outcomes following programme implementation. This aligns with the literature on integration of non-RHD health services including neglected tropical diseases which have previously benefited from integrated programmes, such as schistosomiasis control in Cameroon [12].

A meta-analysis of secondary prophylaxis adherence showed a statistically significant 18% improvement in adherence (p = 0.02) following the introduction of partially-integrated RHD programmes in Cuba, India, and Australia. The programmes in Cuba and India both had an education component which targeted healthcare personnel, while in India this was expanded to teachers and pupils. Both programmes also established a dedicated register to monitor patients with ARF and RHD and to administer prophylaxis. In contrast, the programme in Australia focused on implementing a continuous quality improvement (CQI) strategy for documenting ARF/RHD patients. Therefore, there appears to be multiple channels for improving prophylaxis compliance which may include education about the disease, a register, or improved documentation and care of ARF/RHD patients using a CQI strategy.

None of the programmes were fully integrated, but they did share similarities in the nature and extent of integration into the local health system. The public sector usually took primary responsibility for financing of programmes and for providing clinical care itself, but planning of the RHD programme was never coordinated with the planning of other disease programmes or general health services. Monitoring and evaluation was also not integrated into existing health systems, and demand generation (understood to mean education of at-risk populations) was usually accomplished using a partially integrated approach. The results of this study are in agreement with a similar review which examined the extent and nature of integration for a number of other disease programmes, and found a heterogeneous picture of integration according to the critical health systems functions [16].

Based on existing evidence, we can identify the following best practices for the design of RHD prevention and control programmes. The most effective RHD programmes employ stewardship and governance models that involve a dedicated unit, for example, within a subnational ministry of health office, which is responsible and accountable specifically for RHD. Financing of RHD prevention and treatment interventions should be integrated within general health system resources; external donors wishing to invest in RHD should channel funds through ministries of health to ensure efficient purchasing and strengthening of local systems. In the published literature RHD programme planning has *not* been integrated with planning for other priority health issues; however, it is unlikely that this approach will be desirable in the future, especially for complex and comprehensive RHD programmes that include a variety of activities ranging from primary prevention to surgery.

Published models of care indicate that service delivery is best accomplished through the general primary healthcare system, although targeted case-finding activities may be appropriate in some settings, and when these have been conducted in the past, they have made use of dedicated outreach healthcare workers. As mentioned, monitoring and evaluation of RHD programmes has typically *not* been integrated, and it is not clear how information systems for ARF and RHD should interact with the rest of the health system (since non-integrated registers, for example, may result in superior patient outcomes). Finally, demand generation – understood in this context to mean information, education, and communication – has usually been only partially integrated, such as through dedicated media campaigns and specialised educational activities.

The partially integrated nature of published RHD programmes fits well with the observation that endemic infectious diseases with ‘elimination’ potential may be best addressed eventually through more targeted activities that, as disease incidence declines, can be gradually integrated into the general primary healthcare system [15]. In this way, RHD stands out from other non-communicable diseases, for which there is consensus that vertical approaches are inappropriate [26]. Decision-makers and planners may benefit from thinking about RHD programmes through an infectious disease and elimination framework rather than through a chronic disease framework. This may especially be the case in low-income countries where resources will initially be devoted to primary and secondary prevention rather than cardiac surgery [27].

The findings of this review provide a starting point for the design and implementation of RHD programmes, but they also highlight some major gaps in knowledge, including a lack of clear evidence on the key programme factors that facilitate integration and still deliver good outcomes. For example, it is recommended that monitoring and evaluation of ARF/RHD secondary prevention activities make use of disease registers. There is little evidence that countries have taken disease registers to scale, and existing reports suggest that such registers have not been integrated into general health information and surveillance systems [28]. Further investigation is required into whether these registers would be more effective if they were integrated into health information systems, or rather as parallel information systems.

Comparative research, using prospective quasi-experimental and experimental methods, is needed in order to determine how to optimise the effectiveness of RHD-related health technologies while moving towards fully integrated programme models. Future research should report on the impact of the programme, namely the incidence, prevalence, or mortality, as standard reporting practice. It is also imperative that the local socio-cultural context is seriously considered when designing future RHD prevention and control interventions so that a local evidence-base can be built to directly advise the decision makers of that region or country.

Our review provides a unique and comprehensive analysis of programme integration into health systems while also providing the details of each programme’s inputs, activities, outputs and outcomes. The data documented in this study could provide a starting point for technical experts in endemic countries who are advising ministries of health on the design of their national RHD strategies. That being said, we stress a number of limitations of this review. Firstly, there were date and English language restrictions on searches which may have limited the number of publications found. Secondly, the small number of included studies and heterogeneity among their outcomes meant that sub-group analyses were not possible. Therefore, it was not possible for us to quantify the association between programme integration and health outcomes. It should also be noted that three of the included studies draw from the same overall WHO-developed approach [21,23,29]; they all included elements of health education and secondary prophylaxis. It is unclear whether these three studies included overlapping patient populations. In particular, the study by Iyengar in India was conducted at the same time as the WHO multi-country programme (in which India was a participating country) [21]. There were notable differences in the duration of the studies included (spanning from 2 years to 10 years), with a wide range of data collection dates (mid-1980s to the early 2000s). No large scale clinical trials were analysed and the majority of included studies do not report long term programme impacts.

## Additional Files

The additional files for this article can be found as follows:

10.5334/gh.874.s1 Appendix 1. The Preferred Reporting Items for Systematic Reviews and Meta-analyses (PRISMA) guidelines.

10.5334/gh.874.s2 Appendix 2. Comprehensive search strategy.

10.5334/gh.874.s3 Appendix 3. Integration Score Guide.

10.5334/gh.874.s4 Appendix 4. Description of Programme Inputs and Activities of the Included Studies.

10.5334/gh.874.s5 Appendix 5. Risk of Bias Assessment using the CASP Tool [20].
