# Supplementary material for: Integrating the Prevention and Control of Rheumatic Heart Disease into Country Health Systems: A Systematic Review and Meta-Analysis
Source: Glob Heart. 2020 Sep 14;15(1):62. doi: 10.5334/gh.874 (PMC7500229; doi:10.5334/gh.874)
Supplement: Appendix 2. — Comprehensive search strategy. [file gh-15-1-874-s2.pdf]

Appendix 2: Comprehensive search strategy

	Subject	Search Terms
**PubMed**		
#1	Group A Streptococcus, rheumatic fever, rheumatic heart disease	Pharyngitis[MeSH Terms] OR rheumatic heart disease[MeSH Terms] OR rheumatic fever[MeSH Terms] OR pharyng*[Title/Abstract] OR "sore throat"[Title/Abstract] OR "group A strep*"[Title/Abstract] OR "rheumatic fever"[Title/Abstract] OR "rheumatic heart disease"[Title/Abstract] OR RHD[Title/Abstract]
#2	Health service delivery	(preventative health services[MeSH Terms]) OR delivery of health care, integrated[MeSH Terms]) OR healthcare[Title/Abstract]) OR health care[Title/Abstract]) AND (vertical[Title/Abstract] OR horizontal[Title/Abstract] OR integrated[Title/Abstract] OR coordinat*[Title/Abstract] OR co-ordinat*[Title/Abstract] OR program*[Title/Abstract] OR service*[Title/Abstract])
Search		1 AND 2
Filters: Publication date from 1 January 1990 to 31 December 2017.		
**Scopus**		
#1	Group A Streptococcus, rheumatic fever, rheumatic heart disease	(TITLE-ABS-KEY (pharyngitis) OR TITLE-ABS-KEY ("rheumatic fever") OR TITLE-ABS-KEY ("rheumatic heart disease") OR TITLE-ABS-KEY ("sore throat") OR TITLE-ABS-KEY ("group A strep*") OR TITLE-ABS-KEY (rhd))
#2	Health service delivery	((TITLE-ABS-KEY (delivery) OR TITLE-ABS-KEY (integrated) OR TITLE-ABS-KEY (program*) OR TITLE-ABS-KEY (programme*) OR TITLE-ABS-KEY (service*) OR TITLE-ABS-KEY (horizontal) OR TITLE-ABS-KEY (vertical))) AND ((TITLE-ABS-KEY (health AND care) OR TITLE-ABS-KEY (healthcare)))
Search		1 AND 2
Filters: Publication date from 1 January 1990 to 31 December 2017.		
**EBSCO Host (Africa Wide, CINAHL, and Health Source: Nursing/Academic Edition)**		
#1	Group A Streptococcus, rheumatic fever, rheumatic heart disease	AB "rheumatic heart disease" OR AB "rheumatic fever" OR AB "group A strep*" OR AB pharyng* OR AB "sore throat"
#2	Health service delivery	AB delivery of health care, integrated OR AB health care OR AB healthcare AND AB vertical OR AB horizontal OR AB integrated OR AB coordinat* OR AB co-ordinat* OR AB program* OR AB service*
Search		1 AND 2
Filters: Publication date from 1 January 1990 to 31 December 2017.		
**ISI Web of Science**		
1	Group A Streptococcus, rheumatic fever, rheumatic heart disease	**TOPIC:** ("rheumatic heart disease") *OR* **TOPIC:** ("rheumatic fever") *OR* **TOPIC:** ("group A strep*") *OR* **TOPIC:** (pharyng*) *OR* **TOPIC:** ("sore throat")

2	Health service delivery	(health care) *OR* **TOPIC:** (heath care) AND<br>**TOPIC:** (vertical) *OR* **TOPIC:** (horizontal) *OR* **TOPIC:** (coordinat*) *OR* **TOPIC:** (co-ordinat*) *OR* **TOPIC:** (integrated) *OR* **TOPIC:** (program*) *OR* **TOPIC:**(service*)
Search		1 AND 2

Filters: Publication date from 1 January 1990 to 31 December 2017.
