# Supplementary material for: Integrating the Prevention and Control of Rheumatic Heart Disease into Country Health Systems: A Systematic Review and Meta-Analysis
Source: Glob Heart. 2020 Sep 14;15(1):62. doi: 10.5334/gh.874 (PMC7500229; doi:10.5334/gh.874)
Supplement: Appendix 3. — Integration Score Guide. [file gh-15-1-874-s3.pdf]

**Appendix 3: Integration Score Guide**

| | Fully integrated (s*core = 3)* | Partially integrated (s*core = 2)* | Not integrated *(score = 1)* |
|---|---|---|---|
| **Stewardship and Governance**<br><br>*Oversight and guidance.* | When the governance arrangements for the intervention are the same as those for the general health services or the local or national administrative structures. | The responsibility is shared by the existing general health care system and a specific structure created purposely for the intervention. | When accountability remains exclusively with dedicated specialist entities charged with implementation and management of health interventions, without involvement of the general health care system. |
| **Financing**<br><br>*The pooling of financial resources and the provider-payment methods used to allocate these.* | Funding is provided entirely through the national or regional general health care budget. | For example, where earmarked funding was provided by the United States Agency for International Development (USAID) but channelled through the PHC system. | When financing is provided directly to an intervention and addressing only a particular disease or problem; or directly funded by an external donor. |
| **Planning**<br><br>*Activities, processes and systems for needs assessment, priority setting, and resource allocation.* | If the decision-making in relation to the above three areas is undertaken by institutions/stakeholders who are involved in the same tasks for the general health system. | Decision-making responsibility for planning is retained by those managing the health intervention but involves a range of stakeholders (such as civil society representatives, PHC level, or local/regional/national government) through inclusive groupings. | When the decision-making focused solely on the intervention without consideration of general health care activities. This may include specific national government units at national level. |
| **Service delivery**<br><br>*Structural and organizational dimensions of the programme.* | If their provision is the responsibility of general or multi-purpose health worker. | Where there is shared responsibility for the provision of services between general health workers and the health intervention staff; purpose trained volunteers; when service delivery for a number of interventions is linked. | A number of interventions rely solely on single purpose workers and have no integration with other interventions or general health services. |
| **Monitoring and Evaluation** | Use of shared indicators and establishment of integrated data collection, recording, analysis and reporting systems. | When M&E were undertaken jointly by staff from the regional health services and the control programme. | When M&E is undertaken independently by the sponsor, institution, or volunteers of the implementing organisation. |
| **Demand generation**<br><br>*The use of appropriate financial incentives and monetary support, insurance, or information, education and communication activities designed to change behaviour.* | If mechanisms used to create financial incentives or education and communication activities are provided jointly with the general services or are delivered by primary health care workers. | When education is provided jointly by the targeted programme staff and regional health workers. | Where information campaigns related to health interventions are stand-alone activities, focusing solely on a single problem or disease, and delivered by single-purpose health workers or volunteers. |

*From:* Atun R, de Jongh T, Secci F, et al. Integration of targeted health interventions into health systems: a conceptual framework for analysis. *Health Policy Plan* 2010;25:104–11.http://dx.doi.org/10.1093/heapol/czp055, and Atun R, de Jongh T, Secci F, *et al.* A systematic review of the evidence on integration of targeted health interventions into health systems. *Health Policy Plan* 2010;25:1–14.http://dx.doi.org/10.1093/heapol/czp053.
