# Supplementary material for: Integrating the Prevention and Control of Rheumatic Heart Disease into Country Health Systems: A Systematic Review and Meta-Analysis
Source: Glob Heart. 2020 Sep 14;15(1):62. doi: 10.5334/gh.874 (PMC7500229; doi:10.5334/gh.874)
Supplement: Appendix 4. — Description of Programme Inputs and Activities of the Included Studies. [file gh-15-1-874-s4.pdf]

Appendix 4: Description of Programme Inputs and Activities of the Included Studies

Iyengar and colleagues implemented a secondary prevention programme in Northern India, which was financed by a grant from the WHO Arab Gulf programme for development (AGFUND).[21] The programme included 202 healthcare workers and 773 teachers (in 147 schools), who were trained to recognise suspected cases of ARF and RHD. Educational materials in the form of posters, pamphlets and heart models further delivered information about ARF and how to seek treatment. Through this community-based referral system, suspected cases of ARF and RHD could be sent to the nearest health center for diagnosis and treatment. Standard diagnostics for ARF and RHD were used. A rural health centre-based registry was set up where penicillin injections were administered by pharmacists or nurses. Each registered patient received a secondary prophylaxis card and could receive treatment at any of the four health centres in the area.

The World Health Organization initiated a secondary prevention pilot programme in sixteen different countries. [23] The Ministry of Health in each country was responsible for the operationalisation of the programme, with external funding assistance provided by the AGFUND. Each country employed a national programme manager and a multi-disciplinary advisory committee. A total of 2 138 doctors, 16 480 school teachers, and 5 780 other health personnel received training on ARF and RHD. Group sessions were available for the general public. Further education was disseminated by pamphlets, brochures, posters, radio and television. A central ARF/RHD register was set up in each country to manage patients and deliver secondary prophylaxis through the primary health care system. Guidelines and protocols for care delivery were based on the 1988 WHO Technical Report Series. Case finding efforts focused on the screening of schoolchildren, hospital retrospective case surveys, and referral of suspected or confirmed ARF/RHD cases from hospitals, private clinics or other sources.

Nordet and colleagues report on a 10-year effort to roll out primary and secondary prevention in one province in Cuba (273 933 individuals aged 5-25 in the province were included). [22] The programme was organized and administered by an advisory committee that was part of the provincial health office. Clinical services and educational materials provided as part of the programme were funded through the Ministry of Health and delivered in local hospitals and primary healthcare facilities. Standard medications and diagnostics for pharyngitis, ARF, and RHD were employed. Health information was managed mainly by means of dedicated ARF registers at the provincial teaching hospital and 6 local hospitals. The study did not provide detailed information on systems of (clinical) care delivery but did describe novel healthcare worker and public education campaigns which were intended to increase demand for primary and secondary prevention services.

Ralph and colleagues conducted a secondary prevention programme to promote the implementation of national ARF and RHD management guidelines in Australia. [24] Six primary health care clinics in Aboriginal communities of the Northern Territory (NT) region took part. A project management committee was established comprising the lead project investigators, health service managers, clinicians, staff of the NT RHD Control Program, and staff of RHD Australia.The 3-year project was financed by The National Heart Foundation of Australia, UNICEF Australia Health and the NT Department of Health and Community Services. A continuous quality improvement (CQI) intervention was implemented at the clinics to facilitate the use of the national best-practice ARF/RHD guidelines. The CQI method included two essential components: an RHD clinical audit tool for data collection from clinical records, and the ABCD Systems Assessment Tool (SAT) to assess the clinic systems through discussions with health centre staff about the strengths and weaknesses of their health centre. The CQI was continually revised and improved and annual assessment of ARF/RHD health services were performed.

Kwan and colleagues performed a cross-sectional analysis on a tertiary care programme. [25] The heart failure programme was embedded into existing non-communicable disease (NCD) clinics of two rural hospitals in Rwanda and was supported by Rwanda’s Ministry of Health as well as the non-governmental organisation Partners in Health. The government-subsidised health insurance covered services and medication with modest co-payments charged to individual patients. Further funding from Partners in Health protected patients from health care related out-of-pocket expenses and provided a transportation allowance. One day per week was dedicated to heart failure patient care at the NCD clinics. Each clinic team included 2 nurses and 2 administrative personnel, supervised by generalist physicians. Nurses underwent specialised training on ECG and algorithms for the diagnosis and

management of heart failure. The cause of heart failure was determined following a physical examination and basic echocardiography; diagnosis was guided by pre-defined criteria. Following a diagnosis, each patient would receive a therapeutic plan, 6-week follow ups by nurses, and social support if necessary. Medication was administered daily and directly observed by a community health worker to ensure compliance. The exact diagnostic guideline used for RHD was unclear, but patients were treated with penicillin prophylaxis.
