# Supplementary material for: Integrating the Prevention and Control of Rheumatic Heart Disease into Country Health Systems: A Systematic Review and Meta-Analysis
Source: Glob Heart. 2020 Sep 14;15(1):62. doi: 10.5334/gh.874 (PMC7500229; doi:10.5334/gh.874)
Supplement: Appendix 5. — Risk of Bias Assessment using the CASP Tool [20]. [file gh-15-1-874-s5.pdf]

Appendix 5: Risk of Bias Assessment using the CASP Tool [20]

	Iyengar 1991	Kwan 2013	Nordet 2008	Ralph 2013	WHO 1992
1. Did the trial address a clearly focused issue?	Y	Y	Y	Y	Y
2. Was the cohort recruited in an acceptable way?	Y	Y	Y	Unclear	Y
3. Was the exposure accurately measured to minimise bias?	n/a	n/a	n/a	n/a	n/a
4. Was the outcome accurately measured to minimise bias?	Y	Y	Unclear	Y	Unclear
5. a. Have the authors identified all important confounding factors?<br>b. Have they taken account of the confounding factors in the design and/or analysis?	Y	n/a	n/a	n/a	n/a
6. a. Was the follow up of subjects complete enough?<br>b. was the follow up of subjects long enough?	Y	Y	n/a	n/a	n/a
**Overall risk of bias**	**Low**	**Low**	**Unclear**	**Unclear**	**Unclear**
